# Editorial: Opportunities and Challenges for Drug Discovery From Natural Products in Pharmacotherapy of Neurological Disorders

**DOI:** 10.3389/fnins.2022.936981

**Published:** 2022-05-31

**Authors:** Philippe Jeandet, Vijay Kumar, Devesh Tewari

**Affiliations:** ^1^Faculty of Sciences, University of Reims, Reims, France; ^2^Leiden University Medical Center, Leiden, Netherlands; ^3^Percuros BV, Leiden, Netherlands; ^4^Department of Pharmacognosy and Phytochemistry, Delhi Pharmaceutical Sciences and Research University, New Delhi, India

**Keywords:** bioactivity, metabolomics, molecular targets, natural products, neurodegeneration

The advancement of high-quality data from the human genome and metabolome has led to the exploration of the effects of various natural products in the treatment of neurological and neurodegenerative diseases. Notably, the enormous structural complexity and diversity of scaffolds of natural products are major characteristic features providing great potential for the therapy of these diseases (Tewari et al., 2018; Grinan-Ferré et al., 2021; Uddin et al., 2021). Despite of their tremendous potential, less than 20% of medicinal plants have been investigated for their systemic phytochemical and pharmacological activities. Various natural products can be the potential source for new leading molecules by functioning in line of the “one compound-multiple targets” paradigm. Integration of chemical and metabolomic profiling data, together with the bioactivity data of natural products, can open new possibilities for drug discovery and development to combat various neurological disorders. Modern pharmaceuticals have been developed starting from small molecules extracted from natural resources. Galantamine, a cholinesterase inhibitor, is a classic example of the discovery of small molecules for neuroprotection (Dos Santos et al., 2018). This example, among others, demonstrates that natural products or plant extracts may represent an important pool for generating novel therapeutics. Papers presented in the following issue illustrate these different aspects.

Migraine results in specific headache causing a low but troublesome disability and which is considered as a major public health problem. Many drugs are available for the treatment of migraine but most of them display adverse side effects. In their article, Liu D. et al. investigated the potential of xiongmatang (XMT) extracts used in traditional Chinese medicine to relieve migraine-associated symptoms in a rat model. The XMT extracts represent a mixture composed of two plants, *Ligusticum chuanxiong* and *Gastrodia elata*. A migraine model was established in rats through microinjections of an inflammatory soup consisting of histamine, bradykinin, serotonin and prostaglandin E2. Rats were receiving butanolic and ethyl acetate extracts alone or in complex combinations of decoctions of the two plants. XMT extracts were found to ameliorate the rat behavioral performances through downregulation of factors associated with migraine in the trigeminal nerves. XMT extracts indeed negatively modulate a complex pathway comprising the transient receptor potential vanilloid 1 (TRPV1)/calcitonin-gene-related peptide (CGRP) and its two canonical receptors, CRLR (calcitonin receptor-like receptor) and RMP1 (receptor activity-modifying protein 1). It indeed seems that brain activation of TRPV1 nociceptors located close to the endings of the trigeminal nerve leads to the release of a lot of peptides and particularly CGRP, responsible for the observed neurogenic effects in migraine. XMT extracts by diminishing the TRPV1/CGRP-CRLR-RAMP1 pathway in the trigeminal nerves of rats with migraine could thus ameliorate their behavioral performances.

Lin et al. have used the corticoid-induced neuron-like PC12 cell line as a reliable cellular model for depression to test the potent antidepressant efficacy and the mode of action of hederagenin, a pentacyclic triterpenoid saponin constituent of *Fructus Akebiae* whose herbal extracts are employed in traditional Chinese medicine. The authors postulated that if hederagenin exerts protection against corticoid-induced injury in neurons or neuron-like cells, then this compound may display a significant anti-depression-like activity. Hederagenin at a concentration as low as 0.3 μM was found to prevent mitochondrial potential loss, reduce reactive oxygen species production and apoptosis in corticosteroid-injured PC12 cells. A similar protective effect was also observed in cultures of primary hippocampal neurons. Hederagenin was demonstrated to up-regulate the PI3K/AKT pathway, which is known to play a pivotal role in neuronal cell proliferation and survival, as well as its major downstream targets FoxO3a (Forkhead box class O3a) and GSK3β (glygogen synthase kinase-3-beta), as the application of inhibitors of PI3K and AKT, respectively, suppresses the neuroprotective effects of hederagenin.

The neuroprotective activity of ursolic acid, another pentacyclic triterpene extracted from the skins of fruits or found in herbs and spices, has been described in rats displaying temporal lobe epilepsy, as induced by pilocarpine in the work of Liu K-m. et al. Treatment with ursolic acid was reported to relieve convulsive behavior and cognitive impairment provoked by epilepsy. This compound was able to rescue ectopic migration, aberrant neurogenesis and damage in hippocampal neurons as well as impairing neuroinflammation induced by a prolonged seizure (status epilepticus) and the levels of inflammatory factors such as TNF-α and IL-1β. Ursolic acid was also shown to increase the expression of oxidative stress markers and the mitochondrial oxidative phosphorylation enzyme system (OXPHOS), suggesting it is able to relieve damage linked to oxidation and mitochondrial dysfunction induced by a prolonged seizure. The anti-inflammatory and antioxidant capabilities of ursolic acid thus make it a potent candidate drug for improving epileptic sequelae.

Two azepine-indole alkaloids, nemerosine and fargesine from *Psychotria nemorosa* were investigated in the study of Kirchweger et al. for their ability to modulate serotonergic signaling (human 5-HT_2A_ receptor) and to afford protection against the *in vivo* toxicity of the neurotoxic peptides Aβ and α-synuclein, both involved in Alzheimer (AD) and Parkinson (PD) diseases' pathogeneses, respectively. A primary *in silico* approach using ChEMBL database reported a modulation of the human 5-HT_2A_ receptor by these two alkaloids. Moreover, this ability to regulate the serotonergic pathway was demonstrated to confer a protection against protein toxicity in the worm, *Caenorhabditis elegans*. Nemerosine and fargesine at doses as low as 10 μM were indeed reported to decrease the rate of Aβ-induced paralysis in an Aβ-overexpressing *C. elegans* strain. Similarly, these two compounds reduced α-synuclein toxicity in *C. elegans*, that is, a proteotoxic model mimicking PD synucleopathies in humans. This study thus underlines the potential of these two alkaloids for the treatment of AD and PD and the “one compound-multiple targets” paradigm.

Application of system biology tools is of prime importance to evaluate the therapeutic potential of drugs or natural products. Here, Khanal et al. investigated the efficacy of huperzine A, a natural sesquiterpene alkaloid from *Huperzia* species (*H. serrata*) whose extracts are used in China for the treatment of fever, blood disorders and swelling. Huperzine A exhibits neuroprotective effects and its putative activity as an inhibitor of acetylcholinesterase (AchE) makes it a possible agent for the treatment of AD. Authors reported on the prediction of targets of huperzine A using SwissTargetPrediction, identification of the matching proteins in DisGeNET for AD and further enrichment in STRING to identify the associated pathways. Of the 100 proteins predicted to be targeted with huperzine A, 42 were found to be regulated. Importantly, AchE was primarily targeted with a probability of 1. 49% of the regulated proteins matched using DisGeNET with AD proteins. Moreover, molecular docking reported a high binding affinity of huperzine A with AchE, which was very similar to that of donepezil, an AchE inhibitor used in AD treatment. Interestingly, different pathways regulated by huperzine A were associated with biological targets of AD such as dopamine, serotonine and choline pathways as well as response to β-amyloid.

The papers published in this issue thus illustrate the potential of some natural products, alkaloids and triterpenes and natural extracts for the treatment of neurodegenerative diseases.

## Author Contributions

All authors listed have made a substantial, direct, and intellectual contribution to the work and approved it for publication.

## Conflict of Interest

VK was employed by Percuros BV. The remaining authors declare that the research was conducted in the absence of any commercial or financial relationships that could be construed as a potential conflict of interest.

## Publisher's Note

All claims expressed in this article are solely those of the authors and do not necessarily represent those of their affiliated organizations, or those of the publisher, the editors and the reviewers. Any product that may be evaluated in this article, or claim that may be made by its manufacturer, is not guaranteed or endorsed by the publisher.
